# Editorial: Mechanistic, machine learning and hybrid models of the “other” endocrine regulatory systems in health & disease

**DOI:** 10.3389/fendo.2023.1104746

**Published:** 2023-01-23

**Authors:** Joseph DiStefano, Fady Hannah-Shmouni, Frédérique Clément

**Affiliations:** ^1^ Computer Science & Medicine Samueli School of Engineering and Applied Science Engineering VI, UCLA, Los Angeles, CA, United States; ^2^ Division of Endocrinology, University of British Columbia, Vancouver, BC, Canada; ^3^ Université Paris-Saclay, Inria Centre de recherche lnria de Saclay, Palaiseau, France

**Keywords:** mechanistic model, machine learning model, hybrid mechanistic-machine learning model, artificial neural network model, models for optimizing therapy, gonad system models, thyroid hormone regulation modeling, adrenal regulation modeling

We made this our focus because mathematical modeling of endocrine systems is dominated by models of glucose regulation and dysregulation, the endocrine pancreas, liver, fat and their associated metabolic subsystems. We solicited works on modeling of thyroid, parathyroid, adrenal, gonadal, pituitary and hypothalamus, as well as cytokine regulation of immunomodulating cell-signaling molecules involved in autocrine, paracrine and endocrine signaling.

We succeeded in attracting articles from four areas, seven articles utilizing physiologically-based mechanistic (MEC) and one machine learning (ML) modeling.

MEC modeling is based primarily on mechanistic information (biochemical, biophysical interconnectivity and dynamical couplings), derived from first-principles, and numerical input-output data for quantification. Machine learning (ML) models are based primarily on input-output and features data, typically in much larger quantities, modeled in a different but complementary way, usually using high-level optimization and statistical modeling techniques. Mathematical optimization and simulation methods were major tools of several of the articles, attesting to the importance of engineering and computational methodologies for quantifying and predicting biological and clinical phenomena.

Contributions of the 8 published papers are summarized below. We amplify their main theses, pointing out their novelty and pertinence of their methods and applications within the framework of our Topic.

The paper by Hoermann et al. addresses endocrine regulation teleologically, using a mechanistic model of thyroid hormone (TH) regulation to demonstrate their thesis. Endocrine regulation in the hypothalamic-pituitary-thyroid (HPT) axis is orchestrated by physiological circuits integrating multiple influences, providing responses to overt biological challenges, either defending the homeostatic target hormone range or adapting it to changing conditions. The authors develop their ordinary differential equation (ODE) model for elucidating principles and insights into HPT axis regulation, as a cascade of targeted feedforward/feedback pathways. It includes mechanisms for both homeostasis of free triiodothyronine (FT3), and their adaptation to new levels, when needed, thereby providing optimum resilience in stressful situations.

Wolff et al. address the problem of optimal TH replacement therapy for average human hypothyroid patients, using an optimal control theory/model predictive controller approach to determine dosages that normalize hormone levels, using either monohormone LT4 or combined LT4/LT3 therapy. Their simulation experiments resulted in combined dosages slightly better for most. In patients with rare genetic variants – a particular novelty of this work, fine tuning their simulations suggests one or the other modality depending on the variant.

Cruz-Loya et al. refined and adapted THYROSIM, turning it into a personalized simulation tool, p-THYROSIM – based on gender, BMI and individual hormone levels, to better optimize replacement monohormone LT4 and combined LT4/LT3 dosing, and to better understand how gender and BMI impact thyroid regulation over time. They quantified their new model with 3 large experimental datasets and validated it with a fourth containing data from distinct male and female patients across a wide range of BMIs. They also computed unmeasured residual thyroid function (RTF) – a novelty of this work – from this data and showed that neither BMI nor gender had any effect on RTF predictions – supporting tight TH regulation independent of gender and body size. They showed that p-THYROSIM can provide accurate monotherapies for male and female patients, personalized with their BMIs. Where combination therapy is warranted, results predict that very little (5-7.5 μg) LT3 is needed with LT4 to restore euthyroid levels. As another novelty, nomographs allow estimation of unmeasurable RTFs from patient hormone measurements before treatment.

Li and Androulakis propose a mechanistic model that includes a multicellular suprachiasmatic nucleus (SCN) compartment and HPA axis and investigate properties of circadian timing under photoperiod changes. Its output regulates adrenal glucocorticoid levels, elevated in short photoperiods and associated with peak disease incidence. It predicts that this network is more energy-efficient than the distance-dependent network. Coupling the SCN network by intra-subpopulation and inter-subpopulation forces, they identified negative correlation between robustness and plasticity of the oscillatory network. HPA were strongly entrained to SCN rhythms with a pro-inflammatory high-amplitude glucocorticoid profile. They postulate that these dynamics alterations might govern seasonal disease incidence and symptom severity.

Fazli and Bertram investigate coupled networks of endocrine pituitary cells, examining how local coupling properties between them support large-scale network activity and synchronization of bursting oscillations among the population. Their counterintuitive finding was that structural hubs (cells with extensive couplings to other cells) are typically not functional hubs (cells synchronized with others) – an important step toward understanding pituitary cell networks.

Runvik and Medvedev jointly estimate impulsive time series inputs and continuous system parameters for analyzing luteinizing hormone (LH) secretion rhythms in males. Reconstruction of pulse times from undersampled hormonal time series data is addressed using control theory and “impulsive” ODEs. They also illustrate, with cortisol data, how their method can be applied to other endocrine systems.

Shilo et al. address ovulation, the endpoint of ovarian follicle development and selection from mature follicles. The phenomenological Lacker’s model captures salient features of this complex process. This group proposes a physiologically-based, mechanistic model that signals relative follicle sizes, and reproduces both normal linear growth of dominant follicles, and growth-arrested follicles in polycystic ovary syndrome.

The Razzaq et al. paper reviews deep learning (DL) methods, with focus on assessment of thyroid status and diagnosis of precocious puberty. It provides comprehensive cues for critical analysis of DL approaches in an endocrine context, including dataset building and preprocessing, management of imbalanced/missing data, implementation of neural network architectures, and metrics to assess their accuracy. While already developed DL approaches are efficient in predicting thyroid status from standard lab tests, and assessing biological bone age in establishing precocious puberty, further improvements are expected from embedding multisource information in DL-based endocrine diagnoses.

## Epilogue

The mechanistic and ML modeling techniques used or developed in these works by seasoned and new researchers are highly sophisticated; and their application to endocrine systems has resulted in deep insights or clinically useful results. We solicited but didn’t get any submissions on true hybrid MEC-ML modeling in endocrinology, although we know some groups are working on this integration. MEC models inherently include enormous informational “data” about system connectivity (mechanism), the kind of data that greatly constrain the space of possible solution outputs for given exogenous inputs, initial conditions or internal system perturbations. For this very reason, they typically can be successfully quantified with relatively small input-output numerical data – because informational data about mechanism is inherently equivalent to a great deal of input-output data. In contrast, ML models require very large, often enormously large, input-output data sets to succeed in satisfying their prediction goals.

The two distinct methodologies are being increasingly used together, in complementary ways, to model and solve biomedical problems, e.g. (1–4), but with no clear hybridization unfolding yet. We believe they can be combined more deeply within existing modeling theory, with perhaps *new* applied mathematical theory as well.

It’s a “no-brainer” to imagine how the whole will be much larger than the sum of its parts, given how much information is embedded in MEC models about the systems that generate the data for biosystem ML modeling – information that, with few exceptions, now goes unused in ML modeling.

## Author contributions

JD conceived the Research Topic, chose and organized the efforts of the 2 other co-editors, FC and F-HS, co-wrote the first draft of this Editorial with FC, and finalized it collaboratively with FC and F-HS. All authors contributed to the article and approved the submitted version.
